# Supplementary material for: Actin and CDC-42 contribute to nuclear migration through constricted spaces in C. elegans
Source: Development. 2023 Oct 11;150(19):dev202115. doi: 10.1242/dev.202115 (PMC10617605; doi:10.1242/dev.202115)
Supplement: Supplementary information [file develop-150-202115-s1.pdf]

Table S1. Strains

Strain	Genotype	Reference
Fig. 1		
N2	Bristol, wild type	(Brenner, 1974)
EG1285	*oxIs12[p$_{unc-47}$::gfp] X*	(McIntire et al., 1997)
UD87	*unc-84(n369), oxIs12 X; ycEx60[odr-1::rfp, WRM0617cH07]*	(Chang et al., 2013)
UD279	*cgef-1(yc21), unc-84(n369), oxIs12 X; ycEx60*	(Chang et al., 2013)
UD285	*cgef-1(yc3), unc-84(n369), oxIs12 X; ycEx60*	(Chang et al., 2013)
VC506	*cgef-1(gk261) X*	(Kumfer et al., 2010)
UD923	*cgef-1(gk261), unc-84(n369), oxIs12 X; ycEx60*	This study
UD524	*cgef-1(yc3), unc-84(n369), oxIs12 X; ycEx60; ycEx250[100 ng/ul odr-1::gfp; 5 ng/ul WRM0625dG08; 5 ng/ul WRM0622bA03]*	This study
UD525	*cgef-1(yc3), unc-84(n369), oxIs12 X; ycEx60; ycEx250[100 ng/ul odr-1::gfp; 5 ng/ul WRM0625dG08; 5 ng/ul WRM0622bA03]*	This study
UD526	*cgef-1(yc3), unc-84(n369), oxIs12 X; ycEx60; ycEx250[100 ng/ul odr-1::gfp; 5 ng/ul WRM0625dG08; 5 ng/ul WRM0622bA03]*	This study
UD718	*cgef-1(yc3), unc-84(n369), oxIs12 X; ycEx60; ycEx267[100 ng/uL odr-1; 5 ng/uL WRM0627cD01]*	This study
UD719	*cgef-1(yc3), unc-84(n369), osIs12 X; ycEx60; ycEx268[100 ng/uL odr-1; 5 ng/uL WRM0627cD01]*	This study
UD720	*cgef-1(yc3), unc-84(n369), osIs12 X; ycEx60; ycEx269[100 ng/uL odr-1; 5 ng/uL WRM0627cD01]*	This study
UD721	*cgef-1(yc3), unc-84(n369), oxIs12 X; ycEx60; ycEx270[100 ng/uL odr-1; 5 ng/uL WRM0627cD01]*	This study
Fig. 2		
UD814	*cgef-1b(yc101[S14*]), unc-84(n369), oxIs12 X; ycEx60*	This study
UD815	*cgef-1b(yc102[Δ50 nt in exon 1]), unc-84(n369), oxIs12 X; ycEx60*	This study
UD818	*cgef-1d(yc103[G13*]), unc-84(n369), oxIs12 X; ycEx60*	This study
UD819	*cgef-1d(yc104[Δ49 nt in exon 2]), unc-84(n369), oxIs12 X; ycEx60*	This study
UD822	*cgef-1a,c(yc109[P6*]), unc-84(n369), oxIs12 X; ycEx60*	This study
UD823	*cgef-1a,c(yc110[Δ52 nt exon 1]), unc-84(n369), oxIs12 X; ycEx60*	This study
NK774	*qyEx116 [p$_{cgef-1b}$::GFP + unc-119(+)]*	(Ziel et al., 2009)
NK775	*qyEx117 [pcgef-1a,c::GFP + unc-119(+)].*	(Ziel et al., 2009)
UD725	*qyEx116[p$_{cgef-1b}$::GFP + unc-119(+)]; ycEx244 [odr-1::gfp; p$_{hlh-3}$::nls::tdTomato]*	This study
UD726	*qyEx117[p$_{cgef-1a,c}$::GFP + unc-119(+)]; ycEx244 [odr-1::gfp; p$_{hlh-3}$::nls::tdTomato]*	This study
Fig. 3		
AFS222	*zen-4(cle10) IV*	(Farboud et al., 2019)
UD716	*cdc-42(yc100[degron::GFP11::cdc-42]) II; oxIs12 X; ycEx266[p$_{myo-2}$::mCherry; p$_{hlh-3}$::TIR-1::mRuby]*	This study
UD717	*cdc-42(yc100) II; unc-84(n369), oxIs12 X; ycEx266*	This study

Development • Supplementary information

Strain	Genotype	Reference
UD989	*cdc-42(yc100[degron::GFP11::cdc-42]) II; cgef-1(gk261), unc-84(n369), oxIs12 X; ycEx300[odr-1::GFP, unc-84 (+)]; ycEx266[p_{myo-2}::mCherry; p_{hlh-3}::TIR-1::mRuby]*	This study
Fig. 4		
UD925	*cgef-1(yc3), unc-84(n369), oxIs12 X; ycEx60; ycEx285[100 ng/uL odr-1::gfp; 2 ng/uL pSL884]*	This study
UD926	*cgef-1(yc3), unc-84(n369), oxIs12 X; ycEx60; ycEx286[100 ng/uL odr-1::gfp; 2 ng/uL pSL884]*	This study
UD927	*cgef-1(yc3), unc-84(n369), oxIs12 X; ycEx60; ycEx287[100 ng/uL odr-1::gfp; 2 ng/uL pSL884]*	This study
UD932	*cgef-1(yc3), unc-84(n369), oxIs12 X; ycEx60; ycEx288[100 ng/uL odr-1::gfp; 2 ng/uL pSL885]*	This study
UD933	*cgef-1(yc3), unc-84(n369), oxIs12 X; ycEx60; ycEx289[100 ng/uL odr-1::gfp; 2 ng/uL pSL885]*	This study
UD934	*cgef-1(yc3), unc-84(n369), oxIs12 X; ycEx60; ycEx290[100 ng/uL odr-1::gfp; 2 ng/uL pSL885]*	This study
UD935	*cgef-1(yc3), unc-84(n369), oxIs12 X; ycEx60; ycEx291[100 ng/uL odr-1::gfp; 2 ng/uL pSL886]*	This study
UD936	*cgef-1(yc3), unc-84(n369), oxIs12 X; ycEx60; ycEx292[100 ng/uL odr-1::gfp; 2 ng/uL pSL886]*	This study
UD937	*cgef-1(yc3), unc-84(n369), oxIs12 X; ycEx60; ycEx293[100 ng/uL odr-1::gfp; 2 ng/uL pSL886]*	This study
UD938	*cgef-1(yc3), unc-84(n369), oxIs12 X; ycEx60; ycEx294[100 ng/uL odr-1::gfp; 2 ng/uL pSL887]*	This study
UD939	*cgef-1(yc3), unc-84(n369), oxIs12 X; ycEx60; ycEx295[100 ng/uL odr-1::gfp; 2 ng/uL pSL887]*	This study
UD940	*cgef-1(yc3), unc-84(n369), oxIs12 X; ycEx60; ycEx296[100 ng/uL odr-1::gfp; 2 ng/uL pSL887]*	This study
Fig 5		
BOX409	*par-6(mib30[par-6::degron::egfp-loxp]) I; mibIs49[p_{wrt-2}::TIR-1::tagBFP2-Lox511::tbb-2-3'UTR, IV:5014740-5014802 (cxTi10882 site)]) IV*	(Castiglioni et al., 2020)
BOX607	*pkc-3(mib78[egfp-loxp::degron::pkc-3]) II; mibIs49[p_{wrt-2}::TIR-1::tagBFP2-Lox511::tbb-2-3'UTR, IV:5014740-5014802(cxTi10882 site)]) IV*	(Castiglioni et al., 2020)
UD829	*par-6(mib30[par-6::degron::egfp-loxp]) I; oxIs12 X; ycEx266[p_{myo-2}::mCherry; p_{hlh-3}::TIR-1::mRuby]*	This study
UD830	*par-6(mib30[par-6::degron::egfp-loxp]) I; unc-84(n369), oxIs12 X; ycEx266[p_{myo-2}::mCherry; p_{hlh-3}::TIR-1::mRuby]*	This study
UD831	*pkc-3(mib78[egfp-loxp::aid::pkc-3]) II; oxIs12 X; ycEx266[p_{myo-2}::mCherry; p_{hlh-3}::TIR-1::mRuby]*	This study
UD832	*pkc-3(mib78[egfp-loxp::aid::pkc-3]) II; unc-84(n369), oxIs12 X; ycEx266[p_{myo-2}::mCherry; p_{hlh-3}::TIR-1::mRuby]*	This study
Fig. 6		
DLW29	*dpy-10, wLZ32[p_{sun-1}::TIR-1::mRuby, Cbr-unc-119(+)]; arx-3(lib7[degron::arx-3] III, unc-119(ed3))*	(Zhang et al., 2015)
UD709	*arx-3(lib7[degron::arx-3]) III; oxIs12 X; ycEx253[odr-1::rfp; p_{hlh-3}::TIR-1::mRuby]*	This study
UD710	*arx-3(lib7[degron::arx-3]) III; unc-84(n369), oxIs12 X; ycEx253[odr-1::rfp; p_{hlh-3}::TIR-1::mRuby]*	This study
UD825	*nmy-2(yc111[degron::GFP11::nmy-2]) I; oxIs12 X; ycEx266[p_{myo-2}::mCherry; p_{hlh-3}::TIR-1::mRuby]*	This study
UD826	*nmy-2(yc111[degron::GFP11::nmy-2]) I; unc-84(n369), oxIs12 X; ycEx266[p_{myo-2}::mCherry; p_{hlh-3}::TIR-1::mRuby]*	This study

Development • Supplementary information

Table S2. CRISPR cRNA/Repair Templates

New Alleles	Starting Strain	crRNA	DNA repair template [1, 2]
arx-3(lib7[degron::arx-3] III	wLZ32[psun-1::TIR-1::mRuby, Cbr-unc-119(+)]	AGTTTTCAGgcgATGTCGTC	AAAGTTCCAGTGGAGAACGGAACGCCGGACGACTTCACGAACGCCGCCGCCTCCGGGCCACCGCTTGATTTTTGGCAGGAAACCATCACGTTCTTCCGGTATGATCTCACCGGTGGCCATCCCACAACTTGTGCCTTGGCCGGAGGTTTGGCTGGATCTTTAGGCATcgcCTGAAAACTGACCTAAACTCACAAAAAAT
cdc-42 (yc100[degron::GFP11::cdc-42]) II	ASF222	ACGATCAAGTGCGTCGTCGT	taaagacgtaattttaatacttttattcatttttttttcaggcgaaATGCCTAAAGATCCAGCCAAACCTCCGGCCAAGGCACAAGTTGTGGGATGGCCACCGGTGAGATCATACCGGAAGAACGTGATGGTTTCCTGCCAAAAATCAAGCGGTGGCCCGGAGGCGGCGGCGTTCGTGAAGTCAGGAGCTAGCGGAGCCAGAGATCACATGGTTCTTCATGAATATGTAAATGCAGCTGGAATTACAGGAGGTTCTGGCGGATCAGGAGCTAGCGGAGCCCAGACGATCAAGTGTGTGGTGGTGGGAGATGGAGCTGTCGGTAAAACTTGTCTCCTGATCAGCTATACCAC
cgef-1b (yc101[cgef-1b(S14*)]) X	N2	ACCATGCTGCCGTAGCAGGA	gagagtggagggcctcacgaatcgagtgtttgaaATGCAGGCGGCTCCGTCATGTTATGGCAGCATGGTAGCTTGAACATCAGAAGAAGTCACAACATCCACATTATCGCGGGGACCAATGACCAT
cgef-1d (yc103[cgef-1d(G13* exon 2)]) X	N2	CGAGTTGATGAGATCTTCTC	gaacattttttgcagGAAGAAGTTGAAGCCTCGCGAAATCTCAAAAAAGCTTGAGAAGATCTTATCAACTCGAATGAAGCCGAGgtgaacaagacaacaaaatgacttttcttg
cgef-1a,c (yc109[cgef-1a,c(P6*)]) X	N2	AGGTTCCTGTCGTTCCGGCG	acctaccagcaactctggagcaccagaccgcttttATGAAGCGAACACTTTAAAGGTTCCTGTCGTTCCGGCGCTCAAAGCGTAGGCTCATCACTTCAGTATC
nmy-2 (yc111[degron::GFP11::nmy-2]) I	AFS222	GATGATGTCATtattaccgc	ccgccttttattccatttaacttttcagctaattgattacaacaacagctccagcggtaataATGCCTAAAGATCCAGCCAAACCTCCGGCCAAGGCACAAGTTGTGGGATGGCCACCGGTGAGATCATACCGGAAGAACGTGATGGTTTCCTGCCAAAAATCAAGCGGTGGCCCGGAGGCGGCGGCGTTCGTGAAGTCAGGAGCTAGCGGAGCCAGAGATCACATGGTTCTTCATGAATATGTAAATGCAGCTGGAATTACAGGAGGTTCTGGCGGATCAGGAGCTAGCGGAGCCACATCATCTCGACAAAAAAGATGATGAGATTGACCAATTGAGAGtatgtttcttcaaat
zen-4(cle10) IV	AFS222	UGAUGGAAGCUAACUACCAG	GACCATCTTCAACTCTTCTTACTATGATTCGCCAATACATGATGGAAGCAGACTACCAGCGAGTAGAGATTGCACGTCTCAAAGATTCTCTAAACGACAAGGATG
cgef-1b(yc102[cgef-1b(D50 nt in exon1)]) X	N2	ACCATGCTGCCGTAGCAGGA	**CTGCTACGGCAGCATGGTTGCTAGCACATCAGAAGAAGTCACAACATCCA**
cgef-1d(yc104[cgef-1d(D49 nt in exon 2)]) X	N2	CGAGTTGATGAGATCTTCTC	**GAAGATCTCATCAACTCGAATGAAGCCGAGgtgaacaagacaacaaaat**
cgef-1a,c(yc110[cgef-1a,c(D52 nt in exon 1)) X	N2	AGGTTCCTGTCGTTCCGGCG	**TCCAAGGTTCCTGTCGTTCCGGCGCTCAAAGCGTAGGCTCATCACTTCAGTA**

[1]Lowercase letters indicate nucleotides in intronic regions and uppercase letters indicate nucleotides in the coding regions.

[2]Bold letters indicate nucleotides in deleted region.

Development • Supplementary information

Table S3. Reagents

Reagent Type	Name	Source or reference	Additional Information
Escherichia. coli	OP50	Caenorhabditis Genetics Center (CGC)	
Recombinant DNA reagent	pSL619	(Chang et al., 2013)	$p_{hlh\text{-}3}$::nls::tdTOMATO
Recombinant DNA reagent	pSL814	(Ho et al., 2018)	$p_{hlh\text{-}3}$::TIR-1::mRuby
Recombinant DNA reagent	pSL830	This paper	$p_{hlh\text{-}3}$::GFP(1-10)
Recombinant DNA reagent	pSL884	This paper	$p_{hlh\text{-}3}$::2xHA::cdc-42(G12*V*)
Recombinant DNA reagent	pSL885	This paper	$p_{hlh\text{-}3}$::2xHA::mig-2(G16*V*)
Recombinant DNA reagent	pSL886	This paper	$p_{hlh\text{-}3}$::2xHA::ced-10(G12*V*)
Recombinant DNA reagent	pSL887	This paper	$p_{hlh\text{-}3}$::2xHA::rho-1(G14*V*)
Recombinant DNA reagent	pLZ31	(Zhang et al., 2015)	$p_{eft\text{-}3}$::TIR-1::mRuby
Recombinant DNA reagent	pEL298	(Alan et al., 2013)	$p_{osm\text{-}6}$::cdc-42(G12*V*)::GFP
Recombinant DNA reagent	pEL656	(Norris et al., 2014)	$p_{unc\text{-}25}$::mig-2(G16*V*)::GFP
Recombinant DNA reagent	pEL777	(Norris et al., 2014)	$p_{unc\text{-}25}$::ced-10(G12*V*)::GFP
Recombinant DNA reagent	pEL1021	(Gujar et al., 2019)	$p_{unc\text{-}25}$::rho-1(G14*V*)::GFP
