# Supplementary material for: Actin and CDC-42 contribute to nuclear migration through constricted spaces in C. elegans
Source: Development. 2023 Oct 11;150(19):dev202115. doi: 10.1242/dev.202115 (PMC10617605; doi:10.1242/dev.202115)
Supplement: Supplementary file 1 [file develop-150-202115_review_history.pdf]

Development

Actin and CDC-42 contribute to nuclear migration through constricted spaces in C. elegans

Jamie Ho, Leslie A. Guerrero, Diana E. Libuda, G. W. Gant Luxton and Daniel A. Starr
DOI: 10.1242/dev.202115

Editor: Swathi Arur

Review timeline
Original submission: 22 June 2023
Editorial decision: 26 July 2023
First revision received: 25 August 2023
Accepted: 15 September 2023

Original submission

First decision letter

MS ID#: DEVELOP/2023/202115

MS TITLE: A CDC-42-regulated actin network is necessary for nuclear migration through constricted spaces in C. elegans

AUTHORS: Jamie Ho, Leslie A Guerrero, Diana A Libuda, GW Gant Luxton, and Daniel A Starr

I have now received all the referees' reports on the above manuscript, and have reached a decision. The referees' comments are appended below, or you can access them online: please go to BenchPress and click on the 'Manuscripts with Decisions' queue in the Author Area.

As you will see, the referees express considerable interest in your work, and provided recommendations, most of which can be addressed textually in the manuscript. Reviewer 1 and 3 suggest an experiment with GFP::LMN-1 to follow the P nucleus in support of the hypothesis that these migrate through small spaces, which is worth addressing, since it provides some direct evidence for the observations noted. If you are able to revise the manuscript along the lines suggested, which may involve further experiments, I will be happy to receive a revised version of the manuscript. Your revised paper will be re-reviewed by one or more of the original referees, and acceptance of your manuscript will depend on your addressing satisfactorily the reviewers' major concerns.

Please attend to all of the reviewers' comments and ensure that you clearly highlight all changes made in the revised manuscript. Please avoid using 'Tracked changes' in Word files as these are lost in PDF conversion. I should be grateful if you would also provide a point-by-point response detailing how you have dealt with the points raised by the reviewers in the 'Response to Reviewers' box. If you do not agree with any of their criticisms or suggestions please explain clearly why this is so.

Reviewer 1

Advance summary and potential significance to field

In the submitted work by Ho and colleagues entitled, "A CDC-42-regulated actin network is necessary for nuclear migration through constricted spaces in C. elegans", the authors examine P-cell nuclear migration in C. elegans. Previous studies indicated that during the L1 larval stage P-

© 2023. Published by The Company of Biologists under the terms of the Creative Commons Attribution License (https://creativecommons.org/licenses/by/4.0/).

nuclei migrate between the body wall muscles and epidermis and dramatically constrict—to about 5% of the diameter of the nucleus. Nuclear migration is regulated by the nuclear SUN protein UNC-84 and the KASH protein UNC-83, which together form a LINC complex. UNC-83 located on the outer nuclear envelop interacts with cytoplasmic kinesin-1 and dynein to move nuclei. Loss of unc-83 or unc-84 result in a temperature-sensitive nuclear migration defect in P cell nuclei, where at 25°C, less than half of P-cell nuclei migrate to a ventral position, while at 15°C, ~90% of P-cell nuclei migrate successfully. This observation suggests that another molecular system(s) is also involved with P-cell nuclear migration. Taking advantage of the strengths of genetic analysis in C. elegans, the authors previously identified toca-1 (transducer of Cdc42 dependent actin assembly) in a screen for parallel pathways mediating P-cell nuclear migration. TOCA-1 has domains that interact with Cdc42, WASP, and the plasma membrane, which suggests that Cdc42, WASP and actin polymerization is involved with nuclear migration. In this work, the authors identify an additional gene from the screen, cgef-1 which is a predicted GEF for Cdc42. The authors use rigorous site of action and genetic analysis to provide strong evidence that CGEF-1 acts in the same pathway as CDC-42 and likely acts as a GEF. They provide further evidence that Arp2/3 and NMY-2 also act in this pathway.

Strengths of the paper include the in vivo study of nuclear migration through a confined space, the rigorous genetic analysis, and the use of the elegant cell specific AID degradation system to support genetic studies and show site of action within the P cells. This is a highly rigorous study, and the findings solidify the in vivo importance of Cdc42, Arp2/3 and NMY-2 in nuclear migration for the field. It is also fascinating, as the authors indicate, that there are likely still other pathways for nuclear migration, as knocking out both of pathways (LINC-complex and Cdc42) does not completely block P cell nuclear migration.

Comments for the author

A weakness of the work is the lack of cell biological analysis. There is no visualization of actin or the nuclei themselves during the migration. Do the nuclei get stuck or ever enter the space between the muscle and cuticle. Do the nuclei rupture? This might simply be technically difficult to show. If so, it would be helpful to state that in the manuscript as most readers will be curious.

Specific points to address (these are not major concerns, but ideally should be addressed in some way):

(1) If possible, an analysis of F-actin around the P cell nuclei. If this is not possible, a comment on the technical challenge of this in the discussion would help.

(2) It would be ideal to image the nuclei in the unc-84; cgef-1 double mutant using the GFP::LMN-1 as the authors have previously done (Bone et al., 2016).
Do the nuclei that don't migrate get stuck? Do they fail to ever compress or flatten, like they do during normal migration?

(3) Have the authors considered examining the localization of NMY-2 during P nuclei migration?

(4) In the Discussion it would be helpful to comment on the ability of P cell nuclei to compress and move through an ~200 nm space. In vertebrate cells, it is thought that the limit for nuclear migration is ~10X this diameter (about 2μm, PMID: 23798731). How do the authors reconcile this difference? Do the authors think these nuclei are specially adapted in interesting ways to compress? Are there additional mechanisms for constriction in C. elegans?

(5) The labels in Figure 1A and 1B are far too small for most readers to see.
Please increase their size.

© 2023. Published by The Company of Biologists under the terms of the Creative Commons Attribution License (https://creativecommons.org/licenses/by/4.0/).

Reviewer 2

Advance summary and potential significance to field

In this work, Ho et al. define a pathway involving the CDC-42 small GTPase and the Arp2/3 complex in nuclear migration in C. elegans. It was previously found that the SUN and KASH molecules UNC-84 and UNC-83 are required for ventral migration of the nuclei of the P cells. The P cells extend ventral processes in embryogenesis, and the nuclei migrate through these processes in larval development. As they do so, they must navigate a constricted space between the body wall muscles and the cuticle estimated to be ~5% the width of the P nuclei. Therefore, this is an excellent model of nuclear migration and deformation through confined space, which can be separated from the early cellular process extension which involves distinct molecules (e.g. Rac GTPases). The temperature sensitive nature of unc-84 mutants suggested parallel pathways that control P nucleus migration. An unbiased genome-wide screen for enhancers of unc-84 was conducted and identified the emu mutations. Previous studies identified the toca-1 gene as an emu, suggesting the involvement of the CDC-42 small GTPase. Here, the authors report that the cgef-1 gene is also an emu. cgef-1 encodes a GEF for the CDC-42 small GTPase. The main findings reported are:

1) cgef-1 mutations enhance unc-84, defining a redundant pathway.
2) A nice use of genome editing and promoter expression studies define the d isoform as the relevant isoform in P nuclear migration.
3) Use of the auxin inducible degron system nicely shows that CDC-42 enhances unc-84, and also further enhances unc-84; cgef-1 doubles, suggesting the involvement of other CDC-42 GEFs in this process.
4) A further use of the AID system shows that PAR-6/PKC-3 are not involved downstream of CDC-42, but that the Arp2/3 complex and NMY-2/non-muscle myosin might be.

The authors posit a model by which CGEF-1 activates CDC-42 which induces an actin network used as a substrate by NMY-2 to exert force and cause nuclear deformation and/or migration.

Comments for the author

In general, the manuscript is clear, well-written and demonstrates well the roles of these genes in P migration in parallel to UNC-84 and UNC-83, which link the nucleus to the microtubule network. The data are strong and rigorous, and well-controlled. A reviewer can always suggest additional experiments, but manuscript does not require them. It does a good job of defining these players in this process. This is an important advance in our understanding of the regulation of nuclear migration.

Comments:

1) In mutants that fail to extend the ventral P cell process, the P cells survive and differentiate, resulting in laterally misplaced VD GABA-ergic neurons. However, if the processes extend but the nuclei fail to migrate, the P cells undergo apoptosis. Do the authors have any speculation or data as to why this is? Do PCD mutations block this death, resulting in lateral VD neurons.
2) Is there any evidence of extracellular matrix remodeling by things such as MMPs during nuclear migration? Loosening up the ECM (e.g. hypodermal to cuticle attachments under the muscles) might help the nuclei by open up the confined space.

Reviewer 3

Advance summary and potential significance to field

It can be highly challenging to identify parallel contributors to processes that depend on multiple mechanisms, but the authors successfully leverage C. elegans genetics to accomplish this for nuclear migration. Using a forward genetic screen, the authors identify a CDC-42 GEF (CGEF-1) that works in parallel to the LINC complex to move nuclei through a constricted space in P-cells of the

© 2023. Published by The Company of Biologists under the terms of the Creative Commons Attribution License (https://creativecommons.org/licenses/by/4.0/).

worm. Based on homology to other systems, the authors test and positively demonstrate CDC-42, the Arp2/3-complex, and non-muscle myosin II also function parallel to the LINC complex in P-cell nuclear migration. They point to this as evidence of a CDC-42-regulated actin network driving P-cell nuclear migration. The strong conservation of all these components across animals makes it very plausible this mechanism could recur in other examples of nuclear migration through a constricted space, making it of broad interest. However, as noted below in Suggestions to authors, the authors should address several issues to make their model more convincing.

Comments for the author

The results presented in this manuscript represent a very promising start. However, additional experiments and controls are required to demonstrate the premise of the title, that a CDC-42-regulated actin network is necessary for nuclear migration through constricted spaces in C. elegans.

(1) The only measure of nuclear migration is the number of GFP-positive GABA neuron nuclei counted. It seems extremely likely disruption of other processes could affect this. The authors should demonstrate by at least one other independent method that nuclear migration has failed, as opposed to alternatives such as cell death, failure of differentiation after migration, etc.
(2) While CDC-42, NMY-2, and ARX-3 have been shown to be important to nuclear migration, the authors have not shown they work together in a manner consistent with producing a "CDC-42-regulated actin network". The authors could test this genetically by simultaneously targeting multiple components. If they work in the same pathway, the simple prediction is that they will have no additive effect. Ideally, the authors should also provide some further demonstration of their interaction, such as examining localization of these proteins in absence of the others. Examining actin distribution in the P-cells after P-cell-specific depletion of these factors would also provide powerful support. Without this sort of supporting evidence, these three factors might contribute independently of each other rather than collaborating to organize an actin network.
(3) My impression from the data is that there is still significant nuclear migration even in absence of these factors and the LINC complex, meaning these factors are not “necessary" for P-cell nuclear migration in the absence of LINC complexes. The authors note in the Discussion there may be additional factors/pathways involved. Thus, the putative actin network may be important for and contribute to nuclear migration in absence of LINC complexes, statements that it is "required" should be toned down.

Minor comments:
(1) The main text states yc3 was rescued by WRM0622bA03. However, Figure 2A shows WRM0622bA03 does not cover all of cgef-1, and the figure legend states worms were injected with WRM0622bA03 plus overlapping fosmid WRM0625dG08. Presumably the latter is the case.
(2) Use of cdc-42(G12V) to demonstrate CDC-42 functions downstream of CGEF-1 is not highly convincing. I am not advocating removal of those results, but the authors might have better luck with cdc-42 alleles that mimic GEF activity (i.e., they undergo significant nucleotide exchange in absence of their GEF), such as C18A or F28L.
(3) The legend for Figure 1B states L4 larvae are shown from the lateral side with the anterior to the left and ventral down. However, based on the numbering of nuclei, the bottom right worm seems to face the other direction.
(4) Some factors (e.g., PKC-3) are shown to not affect, or to barely affect nuclear migration when they are targeted for degradation in P-cells. A trivial explanation is that these factors fail to be degraded in the P-cells. As all are GFP-tagged, can the authors examine this directly? If they cannot, this should be acknowledged as a potential limitation.
(5) The statistical analyses of data sets involving three or more groups should be by ANOVA, rather than student’s T-tests.

© 2023. Published by The Company of Biologists under the terms of the Creative Commons Attribution License (https://creativecommons.org/licenses/by/4.0/).

First revision

Author response to reviewers' comments

Dear Swathi,

Thank you for a thorough and prompt review by three reviewers. We are happy that the manuscript was appreciated by all and look forward to hopefully publishing a revised version in *Development*.

Here, we submit a revised manuscript for your consideration. The changes to the text in the revised manuscript are in blue font. Below is our detailed response to the reviewers' comments. The original reviewer comments are in black font. Our responses are in blue font.

We look forward to hearing from you,
Dan Starr (on behalf of all the authors)

Reviewer 1 Advance Summary and Potential Significance to Field:
In the submitted work by Ho and colleagues entitled, "A CDC-42-regulated actin network is necessary for nuclear migration through constricted spaces in C. elegans", the authors examine P-cell nuclear migration in C. elegans. Previous studies indicated that during the L1 larval stage P-nuclei migrate between the body wall muscles and epidermis and dramatically constrict—to about 5% of the diameter of the nucleus. Nuclear migration is regulated by the nuclear SUN protein UNC-84 and the KASH protein UNC-83, which together form a LINC complex. UNC-83 located on the outer nuclear envelop interacts with cytoplasmic kinesin-1 and dynein to move nuclei. Loss of unc-83 or unc-84 result in a temperature-sensitive nuclear migration defect in P cell nuclei, where at 25°C, less than half of P-cell nuclei migrate to a ventral position, while at 15°C, ~90% of P-cell nuclei migrate successfully. This observation suggests that another molecular system(s) is also involved with P-cell nuclear migration. Taking advantage of the strengths of genetic analysis in C. elegans, the authors previously identified toca-1 (transducer of Cdc42 dependent actin assembly) in a screen for parallel pathways mediating P-cell nuclear migration. TOCA-1 has domains that interact with Cdc42, WASP, and the plasma membrane, which suggests that Cdc42, WASP and actin polymerization is involved with nuclear migration. In this work, the authors identify an additional gene from the screen, cgef-1, which is a predicted GEF for Cdc42. The authors use rigorous site of action and genetic analysis to provide strong evidence that CGEF-1 acts in the same pathway as CDC-42 and likely acts as a GEF. They provide further evidence that Arp2/3 and NMY-2 also act in this pathway.

Strengths of the paper include the in vivo study of nuclear migration through a confined space, the rigorous genetic analysis, and the use of the elegant cell specific AID degradation system to support genetic studies and show site of action within the P cells. This is a highly rigorous study, and the findings solidify the in vivo importance of Cdc42, Arp2/3 and NMY-2 in nuclear migration for the field. It is also fascinating, as the authors indicate, that there are likely still other pathways for nuclear migration, as knocking out both of pathways (LINC-complex and Cdc42) does not completely block P cell nuclear migration.
Thank you for this through and accurate summary. We agree with all three reviewers that our genetic approaches are the strength of this manuscript, allowing us to study the *in vivo* importance of CDC-42, Arp2/3 and NMY-2.

Reviewer 1 Comments for the Author:
A weakness of the work is the lack of cell biological analysis. There is no visualization of actin or the nuclei themselves during the migration. Do the nuclei get stuck or ever enter the space between the muscle and cuticle. Do the nuclei rupture? This might simply be technically difficult to show. If so, it would be helpful to state that in the manuscript as most readers will be curious.
We agree that the lack of cell biology analysis is a weakness. Technical difficulties have hampered our efforts to address this deficit. The small size of larval P cells and the propensity for P-cell development to arrest when we attempt to image the process has prevented us from obtaining sample sizes with sufficient statistical power for us to be able to confidently conclude much. This reviewer poses three of the most pertinent questions we have tried to address:

(1) What does the actin cytoskeleton look like during P-cell nuclear migration?

© 2023. Published by The Company of Biologists under the terms of the Creative Commons Attribution License (https://creativecommons.org/licenses/by/4.0/).

We previously published a cursory examination of actin during P-cell nuclear migration using Lifeact:mKate2 or *vab-10-abd::mVenus* expressed from extrachromosomal arrays under the control of a P-cell specific promoter (Bone et al., 2016 *Development*). It was challenging to consistently describe actin in wild-type P cells, making describing the localization of subtle differences in the actin network in mutants problematic. Thus, we do not feel comfortable about making conclusions regarding how the actin cytoskeleton is or is not disrupted in the mutants analyzed here. Therefore, we added the following sentence to the end of Page 2 in the Introduction: "There is an extensive, but poorly defined, actin network in larval P cells (Bone et al., 2016)."

(2) Do nuclei get stuck during P-cell nuclear migration in mutant backgrounds?
We cannot determine if P-cell nuclei get stuck during migration, because often, nuclei stop mid-migration when we try to film them, even in wildtype animals. Given the amount of imaging we have attempted, we are confident that if a significant number of nuclei were getting stuck mid-migration in the mutants, we would have seen that. However, the extent to which they might be delayed is an interesting question that we are unable to answer at this time.

(3) Do nuclei rupture during P-cell nuclear migration?
We have not seen nuclei rupture in wild type or in our limited analyses of the mutants described here. Although our negative result here might be interesting, we do not feel confident saying that there is not a small increase in nuclear rupture events in the mutants analyzed in this wiork.

Specific points to address (these are not major concerns, but ideally should be addressed in some way):

(1) If possible, an analysis of F-actin around the P cell nuclei. If this is not possible, a comment on the technical challenge of this in the discussion would help.
We added the following sentence to the Discussion on pages 6-7: "Further studies are also needed to describe the actin network in mutant backgrounds. There is an extensive actin network in wild-type P cells (Bone et al., 2016) but technical difficulties associated with the small size of P cells and their propensity to arrest development when under the mechanical pressure caused by their compression with a cover slip during imaging have prevented us from analyzing potentially subtle defects in the actin network in mutant backgrounds."

(2) It would be ideal to image the nuclei in the unc-84; cgef-1 double mutant using the GFP::LMN-1 as the authors have previously done (Bone et al., 2016). Do the nuclei that don't migrate get stuck? Do they fail to ever compress or flatten, like they do during normal migration?
The GFP::LMN-1 strain, as reported in Bone et al. (2016) is problematic, causing significant reduced brood size and embryonic lethality. We examined *toca-1* mutants using a nls::tdTomato P-cell nuclear marker and observed nuclei in mid migration, which did flatten out (Chang et al., 2013). It's very difficult to determine if mutant nuclei get "stuck" in a flattened-out state, or if they don't migrate because of of their mechanical compression by the cover slip during imaging.

(3) Have the authors considered examining the localization of NMY-2 during P nuclei migration?
Yes, we actually designed our *nmy-2(AID)* strain to include the 11th strand of split-GFP so we could do this very experiment. Unfortunately, this strategy did not work because the GFP1-10 construct itself had significant background fluorescence alone that obscured any other signal. We have not yet had the opportunity to further pursue this question yet and consider it to be beyond the scope of this manuscript.

(4) In the Discussion it would be helpful to comment on the ability of P cell nuclei to compress and move through an ~200 nm space. In vertebrate cells, it is thought that the limit for nuclear migration is ~10X this diameter (about 2µm, PMID: 23798731). How do the authors reconcile this difference? Do the authors think these nuclei are specially adapted in interesting ways to compress? Are there additional mechanisms for constriction in C. elegans?
This is a very interesting question. However, the differences observed between vertebrate and *C. elegans* nuclear rupture may be a matter of scale differences. Studies in vertebrate cells are on a different scale. They start with nuclei 20 um or larger in diameter. *C. elegans* larval P cells are on the order of 3-4 um in diameter. We don't know the exact width of the space P-cell

© 2023. Published by The Company of Biologists under the terms of the Creative Commons Attribution License (https://creativecommons.org/licenses/by/4.0/).

nuclei traverse. In wild type, P-cell nuclei significantly flatten out (Chang et al 2013 and Bone et al., 2016), but we do not have the axial resolution to be able to accurately measure it. We added the following to the last paragraph of the Discussion on pages 7-8 to address your excellent comments: "There are other pathways that need to be examined in future studies. For example, what role might the nuclear lamina and peripheral heterochromatin play in maintaining P-cell nuclear integrity and compression during their migration? Moreover, how is the extracellular matrix remodeled to provide sufficient space for P-cell nuclear migration to occur? We know that the fibrous organelles are removed right before P-cell nuclear migration (Bone et al., 2016), but determining how wide the constriction that P-cell nuclei migrate through will require the use of correlative light and electron microscopy." Because of the need for CLEM, we feel this issue is outside the scope of the current manuscript.

(5) The labels in Figure 1A and 1B are far too small for most readers to see. Please increase their size.

We have increased the size of the font in Figs 1A-B and thank the reviewer for bringing it to our attention.

Reviewer 2 Advance Summary and Potential Significance to Field:
In this work, Ho et al. define a pathway involving the CDC-42 small GTPase and the Arp2/3 complex in nuclear migration in C. elegans. It was previously found that the SUN and KASH molecules UNC-84 and UNC-83 are required for ventral migration of the nuclei of the P cells. The P cells extend ventral processes in embryogenesis, and the nuclei migrate through these processes in larval development. As they do so, they must navigate a constricted space between the body wall muscles and the cuticle estimated to be ~5% the width of the P nuclei. Therefore, this is an excellent model of nuclear migration and deformation through confined space, which can be separated from the early cellular process extension which involves distinct molecules (e.g. Rac GTPases). The temperature sensitive nature of unc-84 mutants suggested parallel pathways that control P nucleus migration. An unbiased genome-wide screen for enhancers of unc-84 was conducted and identified the emu mutations. Previous studies identified the toca-1 gene as an emu, suggesting the involvement of the CDC-42 small GTPase. Here, the authors report that the cgef-1 gene is also an emu. cgef-1 encodes a GEF for the CDC-42 small GTPase. The main findings reported are:
1)cgef-1 mutations enhance unc-84, defining a redundant pathway.
2)A nice use of genome editing and promoter expression studies define the d isoform as the relevant isoform in P nuclear migration.
3)Use of the auxin inducible degron system nicely shows that CDC-42 enhances unc-84, and also further enhances unc-84; cgef-1 doubles, suggesting the involvement of other CDC-42 GEFs in this process.
4)A further use of the AID system shows that PAR-6/PKC-3 are not involved downstream of CDC-42, but that the Arp2/3 complex and NMY-2/non-muscle myosin might be.
The authors posit a model by which CGEF-1 activates CDC-42 which induces an actin network used as a substrate by NMY-2 to exert force and cause nuclear deformation and/or migration.

Reviewer 2 Comments for the Author:
In general, the manuscript is clear, well-written and demonstrates well the roles of these genes in P migration in parallel to UNC-84 and UNC-83, which link the nucleus to the microtubule network. The data are strong and rigorous, and well-controlled. A reviewer can always suggest additional experiments, but manuscript does not require them. It does a good job of defining these players in this process. This is an important advance in our understanding of the regulation of nuclear migration.
We greatly appreciate these comments. We agree that the strength of this manuscript is the genetics. We tried and certainly wish we had more successes in our cell biology approaches. That being said, we strongly believe that further investigating the cellular mechanisms underlying P-cell nuclear migration will help us better understand this important cellular processes and plan to continue this project in the future. Stay tuned!

Comments:
1)In mutants that fail to extend the ventral P cell process, the P cells survive and differentiate, resulting in laterally misplaced VD GABA-ergic neurons. However, if the processes extend but the nuclei fail to migrate, the P cells undergo apoptosis. Do the authors have any speculation or data as to why this is? Do PCD mutations block this death, resulting in lateral VD neurons.

© 2023. Published by The Company of Biologists under the terms of the Creative Commons Attribution License (https://creativecommons.org/licenses/by/4.0/).

This is an interesting question. We do not have much to speculate other than what we already wrote toward the end of the Discussion about how Rac and Rho mutants block P-cell migration and the P-cells survive in lateral positions (Spencer et al., 2001). Bill Fixsin in the Horvitz lab in his 1985 thesis described the death of P-cells as being different from normal programmed cell death. We added the following sentence to the end of the second paragraph of the Introduction on page 2 of the revised manuscript: "The P-cell death observed in *unc-84(null)* mutatants are morphologically distinct from normal programmed cell death and are not affected by the *ced-3(n717)* mutation (Malone et al., 1999)."

2)Is there any evidence of extracellular matrix remodeling by things such as MMPs during nuclear migration? Loosening up the ECM (e.g. hypodermal to cuticle attachments under the muscles) might help the nuclei by open up the confined space.

This is another fascinating question, but we currently do not know anything about the role of ECM remodeling or MMPs during this process currently. We have added this question to the last paragraph of the Discussion on pages 7-8 of our revised manuscript when talking about future experiments:

"There are other pathways that need to be examined in future studies. For example, what role might the nuclear lamina and peripheral heterochromatin play in maintaining P-cell nuclear integrity and compression during their migration? Moreover, how is the extracellular matrix remodeled to provide sufficient space for P-cell nuclear migration to occur? We know that the fibrous organelles are removed right before P-cell nuclear migration (Bone et al., 2016), but determining how wide the constriction that P-cell nuclei migrate through will require the use of correlative light and electron microscopy."

Reviewer 3 Advance Summary and Potential Significance to Field:

It can be highly challenging to identify parallel contributors to processes that depend on multiple mechanisms, but the authors successfully leverage C. elegans genetics to accomplish this for nuclear migration. Using a forward genetic screen, the authors identify a CDC-42 GEF (CGEF-1) that works in parallel to the LINC complex to move nuclei through a constricted space in P-cells of the worm. Based on homology to other systems, the authors test and positively demonstrate CDC-42, the Arp2/3-complex, and non-muscle myosin II also function parallel to the LINC complex in P-cell nuclear migration. They point to this as evidence of a CDC-42-regulated actin network driving P-cell nuclear migration. The strong conservation of all these components across animals makes it very plausible this mechanism could recur in other examples of nuclear migration through a constricted space, making it of broad interest. However, as noted below in Suggestions to authors, the authors should address several issues to make their model more convincing.

Reviewer 3 Comments for the Author:

The results presented in this manuscript represent a very promising start. However, additional experiments and controls are required to demonstrate the premise of the title, that a CDC-42-regulated actin network is necessary for nuclear migration through constricted spaces in C. elegans.

(1)The only measure of nuclear migration is the number of GFP-positive GABA neuron nuclei counted. It seems extremely likely disruption of other processes could affect this. The authors should demonstrate by at least one other independent method that nuclear migration has failed, as opposed to alternatives such as cell death, failure of differentiation after migration, etc.

We agree with the reviewer that this is an important topic. Therefore we added the following paragraph to the Discussion on page 7 based on our previous findings from Chang et al., 2013: "We conclude that the actin-dependent pathway(s) is(are) important during a narrow window of development in mid L1 when P-cell nuclei migrate. P cells divide within an hour after nuclear migration to the ventral cord to form Pn.p daughtersm, which develop into hypodermal and vulval cells, and Pn.a daughters that are neuroblasts (Sulston & Horvitz 1977). Our enhancer mutant lines were isolated in a primary screen for Egl animals due to defects in the vulval lineages and a secondary screen where we counted GFP-positive GABA neurons (Chang et al., 2013). *cgef-1(yc3)* and *cgef-1(yc21)* disrupt both vulval and neuronal lineages, suggesting that the defect occurs prior to the division of Pn cells to Pn.p and Pn.a. Additionally, in *cgef-1(yc3)* or *cgef-1(yc21)* larvae, there were always the normal number of six P cells present on each lateral side of an embryo or early L1 larva and these P-cells developed normally, narrowing as in wild type until just prior to nuclear migration (Chang et al., 2013). Thus, the critical window for *cgef-1* function is in mid L1,

© 2023. Published by The Company of Biologists under the terms of the Creative Commons Attribution License (https://creativecommons.org/licenses/by/4.0/).

after P cells narrow and before they divide in the ventral cord, which overlaps with the temporal window of P-cell nuclear migration."

(2)While CDC-42, NMY-2, and ARX-3 have been shown to be important to nuclear migration, the authors have not shown they work together in a manner consistent with producing a "CDC-42-regulated actin network". The authors could test this genetically by simultaneously targeting multiple components. If they work in the same pathway, the simple prediction is that they will have no additive effect. Ideally, the authors should also provide some further demonstration of their interaction, such as examining localization of these proteins in absence of the others. Examining actin distribution in the P-cells after P-cell-specific depletion of these factors would also provide powerful support. Without this sort of supporting evidence, these three factors might contribute independently of each other rather than collaborating to organize an actin network.
This is an excellent question. We now clarify in the Discussion toward the bottom of page 6 that we do not yet know if NMY-2 and CDC-42 are in the same pathway or not with the following statement: "…or even if NMY-2 and CDC-42 are in the same or independent pathways."
We also propose a model in the second paragraph of the Discussion where CDC-42 and ARX-3 are in the same pathway based on their known functions in other systems. Furthermore, we added a line discussing the CDC-42, ARX-3 double knockdowns: "While it would be ideal to genetically test whether CDC-42 and ARX-3 are in the same pathway by simultaneously knocking them both out, this would be a difficult experiment to interpret as the AID system only partially knocks down the proteins."
As discussed above, the wild-type baseline for actin structures in L1 P cells is poorly defined, making seeing changes in mutant backgrounds difficult. Thus, we have not looked at actin, NMY-2, or CDC-42 localization in P cells at the time of nuclear migration. While important, these experiments are beyond the scope of this manuscript.

(3)My impression from the data is that there is still significant nuclear migration even in absence of these factors and the LINC complex, meaning these factors are not "necessary" for P-cell nuclear migration in the absence of LINC complexes. The authors note in the Discussion there may be additional factors/pathways involved. Thus, the putative actin network may be important for and contribute to nuclear migration in absence of LINC complexes, statements that it is "required" should be toned down.

We agree that we were overstating by using the words necessary and require. Consequently, we have changed the title from "is necessary for" to "contributes to." We have made similar changes throughout the manuscript.

Minor comments:
(1)The main text states yc3 was rescued by WRM0622bA03. However, Figure 2A shows WRM0622bA03 does not cover all of cgef-1, and the figure legend states worms were injected with WRM0622bA03 plus overlapping fosmid WRM0625dG08. Presumably the latter is the case.

Thanks for catching this. Yes, the latter is the case, both fosmids were injected for the experiment presented in Fig 1F. We have now fixed this in the Results section toward the top of page 4.

(2)Use of cdc-42(G12V) to demonstrate CDC-42 functions downstream of CGEF-1 is not highly convincing. I am not advocating removal of those results, but the authors might have better luck with cdc-42 alleles that mimic GEF activity (i.e., they undergo significant nucleotide exchange in absence of their GEF), such as C18A or F28L.

This is an excellent suggestion and we would like to test mutations like C18A or F28L in the future. However, they are beyond the scope of this manuscript. Therefore, we toned down our interpretation of the *cdc-42(G12V)* result. Specifically, we changed the middle and the last sentences of the third paragraph on page 5. Instead of "concluding" we interpret our results more conservatively. Here are the two new sentences: "Although it was not a complete suppression, this result is consistent with the hypothesis that *cdc-42* is downstream of and activated by *cgef*-1." And "our data suggest that CGEF-1 activates CDC-42 during P-cell nuclear migration." We added the qualifier "partially" to the sentence in the abstract related to this topic. We also changed the title of Figure 4 to "Figure 4: CGEF-1 likely activates CDC-42 during P-cell nuclear migration."

© 2023. Published by The Company of Biologists under the terms of the Creative Commons Attribution License (https://creativecommons.org/licenses/by/4.0/).

(3)The legend for Figure 1B states L4 larvae are shown from the lateral side with the anterior to the left and ventral down. However, based on the numbering of nuclei, the bottom right worm seems to face the other direction.

Thanks for catching this. In fact, a couple were ventral up also. In our revised Fig 1B, we have now flipped them so that they are all in the same orientation with anterior to the left and ventral down.

(4)Some factors (e.g., PKC-3) are shown to not affect, or to barely affect nuclear migration when they are targeted for degradation in P-cells. A trivial explanation is that these factors fail to be degraded in the P-cells. As all are GFP-tagged, can the authors examine this directly? If they cannot, this should be acknowledged as a potential limitation.

This is a limitation of the system. We can't follow degradation via western blots because we are only knocking down the protein in 12 cells. It is also difficult to reliably observe degradation in P-cells because of the presence of background GFP in other tissues. The only way we really know that any of the AID strains are working is when we see a phenotype. We added a sentence to acknowledge this more explicitly. On the bottom of page 5 and top of page 6, we added "A caveat to this experiment is that we do not know how efficient AID of PKC-3 was, although this construct has been successfully used in other tissues (Castiglioni et al., 2020)."

(5)The statistical analyses of data sets involving three or more groups should be by ANOVA, rather than student's T-tests.

Where we were doing multiple t-tests (Figs 1C-E, 2B-C) we are now using one-way ANOVA analyses with Holm-Sidak's corrections for multiple comparisons. This better way of statistics did not change any of our conclusions, although in a couple of places we lost an * on the P values. In Fig 1 C-E, we previously had a few very confusing comparison bars. We have redrawn the brackets to make clearer exactly what we are comparing. Another change is in the brackets to Fig 6A. We now make a single t-test comparison there, with and without the TIR. Finally, In Fig 1 F-G, we are clearer abut our comparison. We have 3-4 independent lines. Within each line, we make a single comparison. Therefore, we can still use the t-tests as we previously did. We did however change the bars to make the comparisons clearer. We edited the figure legends and Materials and Methods to reflect these changes.

<u>Second decision letter</u>

MS ID#: DEVELOP/2023/202115

MS TITLE: A CDC-42-regulated actin network contributes to nuclear migration through constricted spaces in C. elegans

AUTHORS: Jamie Ho, Leslie A Guerrero, Diana A Libuda, GW Gant Luxton, and Daniel A Starr
ARTICLE TYPE: Research Article

I am happy to tell you that your manuscript has been accepted for publication in Development, pending our standard ethics checks.

<u>Reviewer 1</u>

Advance summary and potential significance to field

In the submitted work by Ho and colleagues entitled, "A CDC-42-regulated actin network is necessary for nuclear migration through constricted spaces in C. elegans", the authors examine P-cell nuclear migration in C. elegans. Previous studies indicated that during the L1 larval stage P-nuclei migrate between the body wall muscles and epidermis and dramatically constrict—to about 5% of the diameter of the nucleus. Nuclear migration is regulated by the nuclear SUN protein UNC-84 and the KASH protein UNC-83, which together form a LINC complex.

© 2023. Published by The Company of Biologists under the terms of the Creative Commons Attribution License (https://creativecommons.org/licenses/by/4.0/).

UNC-83 located on the outer nuclear envelop interacts with cytoplasmic kinesin-1 and dynein to move nuclei. Loss of unc-83 or unc-84 result in a temperature-sensitive nuclear migration defect in P cell nuclei, where at 25°C, less than half of P-cell nuclei migrate to a ventral position, while at 15°C, ~90% of P-cell nuclei migrate successfully. This observation suggests that another molecular system(s) is also involved with P-cell nuclear migration. Taking advantage of the strengths of genetic analysis in C. elegans, the authors previously identified toca-1 (transducer of Cdc42 dependent actin assembly) in a screen for parallel pathways mediating P-cell nuclear migration. TOCA-1 has domains that interact with Cdc42, WASP, and the plasma membrane, which suggests that Cdc42, WASP and actin polymerization is involved with nuclear migration. In this work, the authors identify an additional gene from the screen, cgef-1 which is a predicted GEF for Cdc42. The authors use rigorous site of action and genetic analysis to provide strong evidence that CGEF-1 acts in the same pathway as CDC-42 and likely acts as a GEF. They provide further evidence that Arp2/3 and NMY-2 also act in this pathway.

Strengths of the paper include the in vivo study of nuclear migration through a confined space, the rigorous genetic analysis, and the use of the elegant cell specific AID degradation system to support genetic studies and show site of action within the P cells. This is a highly rigorous study, and the findings solidify the in vivo importance of Cdc42, Arp2/3 and NMY-2 in nuclear migration for the field. It is also fascinating, as the authors indicate, that there are likely still other pathways for nuclear migration, as knocking out both of pathways (LINC-complex and Cdc42) does not completely block P cell nuclear migration.

Comments for the author

The authors did a thorough job addressing all concerns about the manuscript.
Congratulation for the outstanding study! I hope in the future that technical challenges can be overcome to image the P-cell nuclear migration event so that the cell biology can pair with the authors deft genetic touch-

Reviewer 2

Advance summary and potential significance to field

The work demonstrates a molecular mechanism that cells use as their niuclei migrate through constricted spaces, linking known nuclear anchoring SUN and KASH molecules with actin via Cdc42 and Arp2/3.

Comments for the author

The authors did a thorough job of addressing reviewer concerns by adding clarifying statements, discussion of additional questions raised by the work, and fixing some typos and figure issues.

Reviewer 3

Advance summary and potential significance to field

It can be highly challenging to identify parallel contributors to processes that depend on multiple mechanisms, but the authors successfully leverage C. elegans genetics to accomplish this for nuclear migration. Using a forward genetic screen, the authors identify a CDC-42 GEF (CGEF-1) that works in parallel to the LINC complex to move nuclei through a constricted space in P-cells of the worm. Based on homology to other systems, the authors test and positively demonstrate CDC-42, the Arp2/3-complex, and non-muscle myosin II also function parallel to the LINC complex in P-cell nuclear migration. The strong conservation of all these components across animals makes it very plausible this mechanism could recur in other examples of nuclear migration through a constricted space, making it of broad interest.

© 2023. Published by The Company of Biologists under the terms of the Creative Commons Attribution License (https://creativecommons.org/licenses/by/4.0/).

Comments for the author

Almost all of my previous concerns have been addressed. My remaining concern is that the authors still show no evidence of a **"CDC-42-regulated"** actin network. While CDC-42 is a major upstream activator of the Arp2/3 complex, it is not the only one known, and there is always the possibility of unknown activators. Moreover, there are cases in which CDC-42 is downstream of actin-dependent trafficking. I appreciate the difficulty the authors note in attempting to simultaneously degrade CDC-42 and ARX-3, and am willing to accept this might not be the best approach. However, in absence of any demonstration F-actin, ARX-3, and NMY-2 function downstream of CDC-42 in this specific context, I disagree with the title's proposition that the authors have demonstrated the actin network is CDC-42-regulated. The Abstract and Discussion are appropriately cautious, but the title still needs to be toned down.

© 2023. Published by The Company of Biologists under the terms of the Creative Commons Attribution License (https://creativecommons.org/licenses/by/4.0/).
